# Neuron identity switches in response to the gradient gene expression pathway

**DOI:** 10.3389/fncel.2025.1536444

**Published:** 2025-02-19

**Authors:** Gustavo Guzmán, Omar Paredes, Rebeca Romo-Vázquez, Hugo Vélez-Pérez, J. Alejandro Morales

**Affiliations:** ^1^Biodigital Innovation Lab, Translational Bioengineering Department, Exact Sciences and Engineering University Center, Universidad de Guadalajara, Guadalajara, Mexico; ^2^Translational Bioengineering Department, Exact Sciences and Engineering University Center, Universidad de Guadalajara, Guadalajara, Mexico

**Keywords:** neuronal continuum, cell identity, glia cells, neurons, axon compartments, glia-to-axon, cellular neighborhoods

## Introduction

Non-static Neuron identity emerges from the complementary synaptic transcriptional architecture shaped by neuron signaling and surrounding non-neuronal cells. When signals from a series of neurons are combined, they form circuits through which information flows, and brain functioning occurs as a superposition of these neural circuits. To ensure proper communication and function, neurons develop complementary phenotypes of axonal projections and electrophysiological behaviors, creating gradients that define brain regions and allocate neurochemical functions (Vogel et al., 2024).

In the mature nervous system, axonal projections extend over long distances and communicate with different well-established regions (Pal et al., 2024) in both peripheral nervous systems (PNS) and central nervous systems (CNS). Information flows inside each axon despite the great distance, resulting in immediate responses.

The mechanism involved in such responses has been described as a soma-centric notion, where the neuronal soma provides total molecular information through the axon (Dalla Costa et al., 2021). However, Nijssen et al. (2018) showed evidence of differences between the axonal transcriptome and the soma transcriptome from Spinal Motor Neurons (MNs).

Several authors suggest that, given these differences, the soma alone may not provide all transcriptomic information. Instead, surrounding glial cells accompanying the axon along its pathway may supply some transcriptomic information. These glial cells play an essential role in proper brain function (Dalla Costa et al., 2021; Giuditta et al., 2008).

The described glia-to-axon relationship becomes particularly significant in the CNS, where different brain regions have distinct molecular, cellular, and functional characteristics (Vogel et al., 2024; Siletti et al., 2023). As axons extend through various brain areas, their transcriptome profiles differ from their soma's (Nijssen et al., 2018). This molecular variation suggests that axons adapt their transcriptomic identity to match their local cellular environment as they traverse different brain regions. The local glial cells provide specific transcriptional resources to the axon in each area. We term this space-dependent, dynamic identity adaptation of neuronal axons the “neuronal continuum.”

## Axonal identity differ from neuronal soma

Modern neuroscience has produced high-resolution cell classifications of the brain based on expression profiles from single-cell sequencing techniques such as Single-Nucleus RNA sequencing (snRNA-seq) (Siletti et al., 2023). This technique focuses on the transcriptional cell identity from the cell nuclei (Hodge et al., 2019). These atlases provide us with CNS cellular cartography for most brain regions of some species (Siletti et al., 2023; Yao et al., 2023; Chen et al., 2023), and they typically report two main groups of neurons—glutamatergic and GABAergic—together with a third non-neuronal cell lineage known as glia, which plays critical regulatory roles throughout the brain (Liu et al., 2023).

Different cognitive functions are region-associated and require specific configurations of axon-dendrite junctions (Dalla Costa et al., 2021), which determine how information flows. These physical pathways, where dendrite and axon morphology can change, are crucial for neural communication. Recent research has shown that changes in these junctions often accompany regulation in local gene expression (Gao et al., 2023).

Nijssen et al. (2018), developed a method for sequencing the transcriptome from a single axon, similar to snRNA-seq, called Axon-seq. They sequenced axons from MNs, finding that the axonal transcriptomic profile differs from its soma in a single neuron. More interestingly, a unique transcription factor signature was found in distal axons that was not found in any of the soma reported, leading us to some questions: Who is responsible for this transcriptomic identity switch, and why does this happen?

## Glia-to-axon communication

Glial cells constitute half of the cell population in the mammalian nervous system. The glia-to-neuron ratio varies across brain structures and species (Liu et al., 2023). They are classified into different types: astrocytes, microglia, synantocytes (Tizabi et al., 2024), oligodendrocytes, and Schwann cells, the last two being the myelin cells of the nervous system. Similar scenarios exist in PNS and the CNS, where glial cells and neurons maintain a close relationship. Glial cells residing at neuronal junctions or within axonal segments play crucial roles in providing fundamental communication support for neuronal survival (Liu et al., 2023).

Recent findings have highlighted the fundamental contribution of glial cells to brain function. Glial cells show shifting characteristics in the myelin sheaths (Xin and Chan, 2020). Glial cells are also very diverse, with varying and specific subtypes and proportions of cellular neighborhoods along brain regions; each neighborhood has its own types of neurons and glia (Siletti et al., 2023).

Giuditta et al. (2008) described glia-to-axon mechanisms from direct evidence with the squid giant axon, showing that beyond simple communication between the glia surrounding the soma/axon, the glia provides the molecular content to the PNS axon. Studies have also reported glia-to-axon communication with Schwann cells that interact with axons to support and deliver genetic material and machinery (Das et al., 2021). Court et al. (2008) and later, Cada and Mizuno (2024), demonstrated the flow of ribosomes from the glia into the axon. Recently, other authors (Krämer-Albers and Werner, 2023) have explained the glia-to-axon communication mechanism between oligodendrocytes and axons, describing the mechanism of exosome (cargo with molecular information) exchange oligodendrocyte-to-axon.

## Neuron and glia transcription

Besides PNS neurons, where axons can reach up to a meter in humans (Twiss and Fainzilber, 2009), CNS axons can travel long distances through the layers and regions of the brain. The frontotemporal arcuate fasciculus (AF) is a well-studied pathway that connects different brain regions. This white matter bundle involved in language processing is 4–5 cm long (Basile et al., 2024).

Despite the long distance, a soma-centric delivery was the primary proposed mechanism for mRNA transport (Dalla Costa et al., 2021). However, Twiss and Fainzilber (2009) reported an anterograde mRNA transport rate of only 16 mm/h, suggesting that relying solely on the nuclear transcription mechanism or any other mechanisms from nuclei only as mRNA synthesis is insufficient due to anterograde and retrograde pathway that information needed to travel. These observations highlight the limitations of single transcriptional machinery in the nucleus to support long axons.

## Gradients and scales

Gene expression gradients were initially described in a rostral-caudal direction (Fornito et al., 2019). Lau et al. (2021) shows a gradual decay of gene expression according to physical distance in the mouse cortex. Furthermore, Vogel et al. (2024), described three principal axes of gene expression gradients aligned with the brain's anatomical architecture.

In addition, snRNA-seq studies showed continuity for major neuronal populations in the adult human and mouse brains (Siletti et al., 2023; Hodge et al., 2019), describing gradients of local cell type identity within each brain region.

Current neuroscience explores the brain across multiple scales (micro-meso-macro scales), each defined by its informational unit. For example, at the mesoscale, the unit would be a set of neighboring cells with locally similar gene expression. From this shared expression, other characteristics emerge, i.e., morphology, function, and interconnectivity (Poulin et al., 2016). Each cellular neighborhood is fuzzily self-defined in a physical space in the brain, and neighborhood interconnectivity will require those boundaries to shift dynamically their gene expression in microgradients. Individual axons whose segments cross different neighborhoods will require multiple local transcriptional sources to thrive.

## Neuronal continuum

Since continuity has been reported across different scales, it is plausible that this phenomenon also occurs within individual neurons. Each brain region is molecularly, cellularly, and functionally distinct and has gene expression patterns necessary for proper cerebral function.

**Figure 1:**
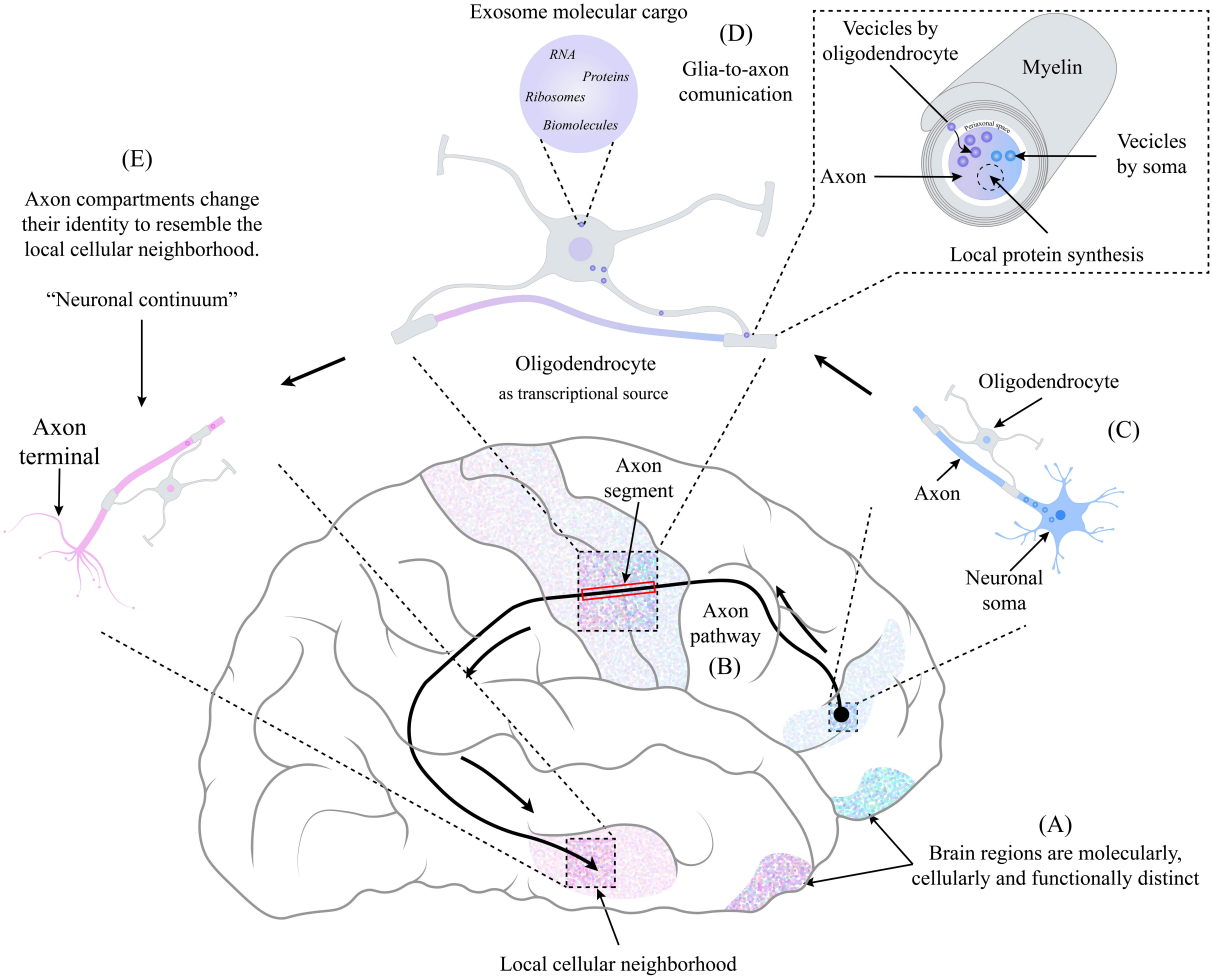
The identity of an axonal compartment switches in the function of the physical location through which it moves, resembling the identity of the local cellular neighborhood. **(A)** Each brain region is molecularly, cellularly, and functionally distinct. **(B)** Axonal pathways may be longer. The frontotemporal arcuate fasciculus is a well-studied pathway connecting different brain regions. **(C)** At the beginning of the axonal path, the neuronal body is in a specific brain region as its axon begins its pathway. On the way, the axon is accompanied by oligodendrocytes (and other glial cells), and a very close relationship is maintained. **(D)** Due to their relationship, a glia-to-axon communication occurs, where, through the mechanism of exosome transfer (Krämer-Albers and Werner, 2023), the oligodendrocyte provides the necessary information and molecular machinery to the axonal segment, which depends on the cellular neighborhood in which it is located. Giving rise to the local synthesis of proteins that, combined with the molecular content coming from the soma (Dalla Costa et al., 2021), produces a switch of identity resembling the cellular neighborhood. **(E)** at the end of the axonal pathway, each axonal compartment has an identity distinct from the neuronal soma and other axonal compartments, creating a non-static identity space-dependent that we term “Neuronal Continuum.”

While information flows along the axons that travel long distances, they visit different brain regions and change their morphology and gene expression. Each axonal segment acquires a distinct transcriptional identity from the soma, combining the nuclear molecular content with the one supplied by the surrounding glial cells in their neighborhood (Farias et al., 2020). Such neuronal continuum means neurons do not show a single identity but a continuous molecular profile collectively established by axonal segments and the soma, dependent on the cellular neighborhood (Figure 1).

We propose that oligodendrocytes, and secondarily other glial cells, serve as multiple transcriptional sources glia-to-axon, providing molecular machinery and content to axonal compartments in the CNS, similar to the role of Schwann cells in the PNS. Recent findings demonstrate that oligodendrocytes play a leading role in regulating neural synapse development, synaptic transmission, and plasticity (Liu et al., 2023; Xin and Chan, 2020).

## Discussion

The neuronal continuum impacts myelin-associated diseases and other neuronal disorders. Nijssen et al. (2018) compared the axonal transcriptomic profile from healthy and amyotrophic lateral sclerosis MNs, demonstrating a differential expression of 121 mRNAs necessary for the property neuron function. Also, oligodendrocyte heterogeneity is implicated in conditions such as Multiple Sclerosis. In this typical demyelination disease, it was shown that differences in oligodendrocyte subtypes between control and patients could contribute to inflammation (Jäkel et al., 2019), possibly due to a disruption of molecular supply at certain axon segments derived from missing oligodendrocyte subtypes. Recent studies have also reported that myelin-related cells impact conditions commonly associated with neuronal disorders (Murdock and Tsai, 2023). By acknowledging the essential role of glia-to-axon for spatially dependent molecular resources, we can better understand the complex interplay between neurons and glia in health and disease.

The traditional view of neurons possessing a fixed identity defined solely by their intrinsic properties is being challenged by emerging evidence of a neuronal continuum. Recognizing that neuron identity is not static but spatially dynamic, influenced by surrounding glial cells within a cellular neighborhood, offers a new understanding of how neuronal identity is established and maintained. By exploring the neuronal continuum, we can improve our understanding of various conditions, such as axon-related diseases, to design novel interventions for neurodegenerative diseases. It also opens new avenues for research into neural development, regeneration, and therapeutic strategies targeting glial-neuronal interactions.
